# Emotional and Spontaneous Locomotor Behaviors Related to cerebellar Daidzein-dependent TrkB Expression Changes in Obese Hamsters

**DOI:** 10.1007/s12311-022-01432-1

**Published:** 2022-07-06

**Authors:** Raffaella Alò, Gilda Fazzari, Merylin Zizza, Ennio Avolio, Anna Di Vito, Ilaria Olvito, Rosalinda Bruno, Marcello Canonaco, Rosa Maria Facciolo

**Affiliations:** 1 Comparative Neuroanatomy Laboratory, Biology, Ecology & Earth Science Department (DiBEST), University of Calabria, Arcavacata Di Rende, Ponte Pietro Bucci 4B, 87030 Cosenza, Italy; 2 Experimental and Clinical Medicine Department, Molecular Oncology Laboratory, University Magna Graecia of Catanzaro, 88100 Catanzaro, Italy; 3 Health Center Srl, Biomedical and Nutritional Center, via Sabotino 66, 87100 Cosenza, Italy; 4 Department of Pharmacy and Science of Health and Nutrition, Edificio Polifunzionale, University of Calabria, Arcavacata Di Rende, 87036 Cosenza, Italy

**Keywords:** Obesity, Neuroprotection, Anxiety, Crebellum, TrkB, Polyphenols

## Abstract

Current evidence supports the beneficial role of phytoestrogens in metabolic diseases, but their influences on spontaneous motor and anxiety behaviors plus neuroprotective effects have still not been completely elucidated. With the present study, neuro-behavioral activities were correlated to daidzein (DZ)-dependent expression changes of a high affinity catalytic receptor for several neurotrophins, and namely tropomyosin-related kinase B receptor (TrkB) in the cerebellar cortex of high-fat diet (HFD) hamsters (*Mesocricetus auratus*). Indeed, these changes appear to be tightly linked to altered plasma lipid profiles as shown by reduced low-density lipoproteins plus total cholesterol levels in DZ-treated obesity hamsters accounting for increased spontaneous locomotor together with diminished anxiety activities in novel cage (NCT) and light/dark box (LDT) tests. For this latter case, the anxiolytic-like hamsters spent more time in the light compartment, which was retained the aversive area of the LDT box. As for the evaluation of the neurotrophin receptor site, significantly elevated TrkB levels were also detected, for the first time, in the cerebellum of obese hamsters treated with DZ. In this condition, such a treatment widely led to an overall improvement of HFD-induced neurodegeneration damages, above all in the Purkinje and granular layers of the cerebellum. In this context, the notably active TrkB signaling events occurring in a DZ-dependent manner may turn out to be a key neuroprotective element capable of restoring normal emotional and spontaneously linked locomotor behaviors regulated by cerebellar cortical areas especially in obesity-related conditions.

## Introduction 

The growing increase regarding the incidence of obesity has attracted scientific interests aimed towards the identification of novel natural compounds capable of modulating food intake that may constitute a valid pharmacological alternative for the treatment of obesity and metabolic syndromes. From recent studies, it seems that dietary supplements are becoming more and more appropriate for the management of obesity and other metabolic disorders as suggested by some polyphenolic extracts reducing the accumulation of intracellular lipids, oxidative stress, and inflammation through the regulation of different metabolic pathways [1, 2]. Of the different bioactive isoflavones, daidzein (DZ), which is abundant in soy, has shown to exert beneficial effects on adiposity with a subsequent reduction of adipose tissue deposition stimulating lipolysis via the activation of the hormone-sensitive lipase [3]. This isoflavone accounts for improved mood, behavior, and cognitive functions especially in anxious plus depressed conditions [4, 5]. Interestingly, DZ has shown to attenuate cerebral inflammation and oxidative stress [6], along with influencing the intestinal bacterial composition [7], as well as being involved with the induction of apoptotic activity of cancer cell [8]. These polyphenols are also known to actively exert a protective role in high-fat diet (HFD) conditions, which represents one of the major health concern responsible for neuronal complications in telencephalon plus hypothalamic areas [9] as suggested by smaller cortical thickness and reduction of gray matter volume under such conditions [10]. As a consequence, polyphenols tend to promote an important functional role especially when they are involved with neuroprotective measures following neurodegenerative alterations or with neuronal dysfunctions that are typical of metabolic obesity states [11].

It has been suggested that neuronal factors, such as neurotrophins, are involved not only with the balance of lipid and glucose levels but are also for the regulation of energy expenditure and cardiovascular homeostasis [12, 13]. Among these, the brain-derived neurotrophic factor (BDNF) and its catalytic receptor tropomyosin-related kinase B receptor (TrkB), which are widely expressed throughout the brain, seem to play a crucial role on synaptic transmission and plasticity events during both the developmental and adulthood stages [14, 15]. Altered expression levels of neurotrophins and Trk receptors resulted to be modified by the ingestion of flavonoids. It appears that the activation of MAP kinase plus PI3 kinase intracellular signaling pathways [16] and the incremented synthesis of NGF together with BDNF improve not only cognitive events [17, 18] but also motor disorders [19].

Based on the above indications, behavioral effects of HFD enriched with simple carbohydrates and lipids ± the phytoestrogen DZ were assessed to evaluate exploratory, emotional plus locomotor activities of the Syrian golden hamster (*Mesocricetus auratus*) when exposed to light/dark test (LDT) and novel cage test (NCT). Amino cupric silver stain (ACS) techniques were applied to determine neuronal damages in the different layers of the hamster cerebellum cortex. Additionally, TrkB expression activities were estimated in the cerebellum cortex of HFD hamsters with respect to animals that received a standard diet aimed to establish the potential role of cerebellum TrkB on emotional and motor performances in HFD hamsters treated with DZ. The selection of this brain area was based on recent evidences corroborating that obesity and body mass are tightly related to a lower volume of brain gray matter, especially in areas like the cerebellum that play a key role in executing motor activities [20]. Indeed, this region, which is involved with the coordination of regulatory processes, sequencing, plus interhemispheric exchanges [21], the control of cognition [22] and sensory emotional activities [23, 24] may turn out to be a pivotal neuronal station for the recovery of behavioral performances in obesity-related disorders.

## Materials and Methods

### Animals

Adult male Syrian golden hamsters (*Mesocricetus auratus*; Envigo Laboratories, Udine-Italy, *n* = 50) weighing 120–180 g were housed individually under a temperature-controlled environment (22–24 °C), at a 14-h light/10-h dark cycle (lights on 07:00 a.m.) and humidity (60%) conditions with food and water available ad libitum. After 1 week of acclimatization, they were randomly assigned to the following groups: (1) group I (control group, CTRL; *n* = 18): hamsters were fed with the standard laboratory rodent chow diet some for 14 (*n* = 6), others for 16 consecutive weeks (*n* = 12); (2) group II (HFD group, *n* = 16), of which some were fed with HFD (60% of calories from fat; analytical components: crude protein 23.00%, crude oils and fats 34.00%, crude fiber 5.00%, crude ash 5.50%, carbohydrates 27.3%; Envigo Laboratories, Udine-Italy) for 14 consecutive weeks (*n* = 6), while others for 16 consecutive weeks (*n* = 10) to induce obesity; (3) group III (HFD + DZ group, *n* = 16): some hamsters were fed with HFD for 14 weeks while others for 16 consecutive weeks, in which DZ (200 mg/kg diet; 98% DZ; Santa Cruz Biotechnology) was added during the last 2 weeks (*n* = 6, HFD + DZ; 2 w) or 4 weeks (n = 10, HFD + DZ; 4 w) of treatment [25]. The effects of the hyperlipidic diet on body weight of each hamster were monitored weekly to assure the state of obesity. The effects of only DZ group were studied in a previous work [5] and so was not handled in the present paper.

After the last behavioral session, hamsters were sacrificed by decapitation and blood was collected (hamsters fasted overnight prior to the blood collection), for the evaluation of some serological markers (i.e., total cholesterol, TC; low-density lipoprotein cholesterol, LDL, and high-density lipoprotein cholesterol, HDL). The concentration was determined using enzymatic colorimetric methods (CHOD-PAP; GPO-PAP; GOD POD) according to manufacturer’s protocol (Biogramma srl; Biotecnica Instruments, Rome-Italy) plus modifications.

Animal maintenance and experimental procedures were carried out in compliance with ethical provisions for Care and Use of Laboratory Animals reported in the legislative law *n*° 26 (04–03-2014) and authorized by the National Committee of the Italian Ministry of Health. Efforts were made to minimize animal suffering and reduce the number of experiments.

### Behavioral Tests

After the diet, all animals individually were subjected to light/dark (LDT) and novel cage (NCT) tests. The behavioral procedures were conducted during the light phase (between 2:00 and 6:00 p.m.) in a sound-isolated room and video-taped via high resolution Waterproof Action Camera (DBPOWER- SJ4000 SPORTS HD DV) mounted vertically above the chamber. The data were analyzed with the specific software EthoLog (version 2.2.5; Visual Basic, São Paulo, Brazil). At the end of each test, the apparatus was cleaned with 0.1% acetic acid to remove all traces of previously tested animals.

#### Light–Dark Test (LDT)

LDT apparatus consists of a box with two compartments: a first arena composed of a small and dark opaque familiar compartment (16 × 16 × 16 cm) containing a black glass while the second arena containing a large translucent and white illuminated compartment (25 × 25 × 30 cm), considered the unfamiliar environment. The compartments were connected by a communicating door (7 × 7 cm), which allowed hamsters to freely move from one compartment to the other.

Prior to the behavioral observations, animals were subjected to a brief period of habituation in the LDT apparatus; then, the hamster was placed in the center of the light compartment with their head opposite to the opening and was allowed to explore the two compartments for 300 seconds (sec). During this period, the following behavioral measures were recorded: light permanence (time spent in the light compartment), dark permanence (time spent in the dark compartment), latency to dark (the first latency to enter the dark compartment), rearing (time spent standing on hind legs), and risk assessment (time in which head and/or body extends from the dark to the light compartment); number of transitions between compartments and risk assessment-number. The advantage of using this apparatus is linked to the presence of an unfamiliar environment so that the hamster is placed in front of a natural conflict deriving from the necessity to explore a novel environment (spontaneous exploratory behavior) and the innate aversion to an open and brightly illuminated arena. In the latter case, it was the presence of light and not darkness that were responsible for the induction of stress-like conditions for hamsters, as shown by the percentage of time spent in the dark compartment. Moreover, the exploratory behavior in the light compartment was also used as an indicator of anxiety. Similarly, the number of crossings between compartments provide an estimation of the anxiety level if measured without changes of spontaneous locomotor activity [26].

#### Novel Cage Test (NCT)

To investigate spontaneous locomotor activity [27], hamsters were placed individually at the beginning of the test in the center of a novel, clean, transparent plexiglass cage (50 × 50 × 30 cm), under light intensity of approximately 200 lx without water and food. The behavior was recorded with a video for 5 min (300 sec) and categorized as mobility (movement in the cage), rearing (standing on hind limbs), wall-rearing (standing on hind limbs and touching the walls of the cage with forelimbs), sniffing (sniffing sawdust), digging (digging sawdust with forelimbs, often kicking it away with hind limbs), grooming (licking own fur, sometimes using forepaws, passing them over the nose with a series of brief, horizontal movements), and climbing (escape behavior). Following the final behavioral session, hamsters were anesthetized, and blood plus brains were taken and processed for biochemical analysis.

### Protein Extraction and Western Blotting

The cerebellum was removed and stored at − 80 °C until western blotting (*n* = 36). Frozen cerebellar tissues (*n* = 6 from each experimental group) were homogenized on ice in a lysis buffer (20 mM Tris–HCl, pH 7.6, 15 mM Triton X-100, 10% glycerol, 2 mM EDTA) containing a cocktail of protease (Roche) and phosphatase (Sigma) inhibitors. Samples were centrifuged at 13,000 rpm at 4 °C for 20 min and the supernatant was collected, stored at − 80 °C for future immunoblotting analyses. Protein concentrations were determined using the Bradford protein assay (Bio-Rad, Hercules, CA). Equal amounts of protein per sample (20 µg) were separated by electrophoresis on 8% and 10% Tris–glycine gels with 4% stacking gels; then, they were transferred to nitrocellulose membranes and the membranes were blocked with 5% serum albumin for 1 h. The nitrocellulose membranes were incubated overnight at 4 °C with the following antibodies: rabbit monoclonal anti TrkB (80E3; EuroClone, Milano, Italy) and goat polyclonal anti β-actin antibody (C-20; sc-7396, 1:2000, Santa Cruz Biotechnology). Blots were then incubated with the appropriate horseradish peroxidase (HRP) conjugated goat anti-rabbit IgG (HRP-linked antibody; EuroClone, Milano, Italy) secondary antibody and developed using enhanced chemiluminescence detection system (Bio-Rad Laboratories). Optical density (O.D.) was determined using the NIH Image J software. Protein expression was normalized to the β -actin protein.

### Cerebellar Histological Analyses

#### Amino Cupric Silver (ACS) Stain

In order to evaluate the argyrophilic reaction, an ACS staining technique was used to determine cerebellar fields that featured advanced damaged cell bodies, dendrites, axons, and terminals of both HFD and DZ treated groups subjected to a diet for 16 consecutive weeks. For this part, cerebral sections (30 µm) were obtained from each of the above experimental groups: CTRL (*n* = 4), HFD (*n* = 4) and HFD ± DZ (*n* = 4), at an interval of 240 µm for ACS procedures as previously described [28]. The estimation of damaged limbic neuronal fields required the volume (defined as Vref) of three layers of cerebellar cortex, and namely the molecular layer (ML), Purkinje layer (PL), and granular layer (GL) by using the following formula:

$$Ns=[ \sum (\mathrm{N}/\mathrm{vsetion })/n] \times \mathrm{V}ref$$

In this calculation, *Ns* represents the number of stained damaged neurons; *N* is the number of damaged neurons in a single section; Vsection is the volume of a single section; *n* is the number of sections; Vref is the total volume of above brain regions.

For general histological observation, some cerebellar sections (16 µm) of CTRL animals (*n* = 2) were stained with Nissl dye.

### Statistical Analysis

The behavioral data and the number of degenerating cells were expressed in % ± S.E.M. while molecular biochemical data were expressed as OD ± S.E.M. Data of HFD hamsters were evaluated and compared with CTRLs (*) and to HFD (letters) using a two-way ANOVA followed by post hoc Newman-Keuls multiple range test when *p* < 0.05; *, ^a^ *p* < 0.05; **, ^b^ *p* < 0.01; ***, ^c^ *p* < 0.001.

## Results

### Effects of DZ on Anxiety-like Response in LDT

**Fig. 1:**
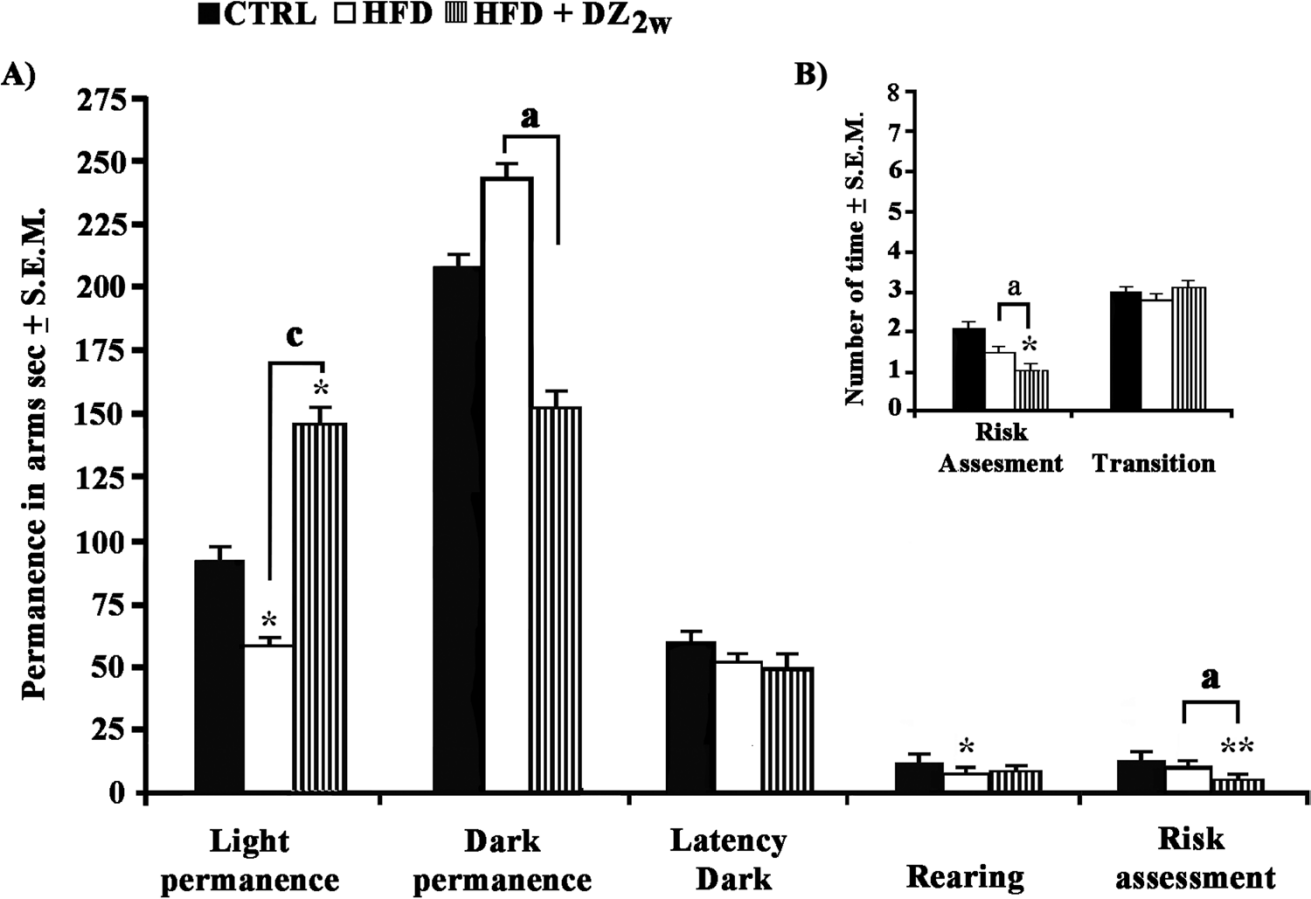
Evaluation of the anxiolytic activity of DZ using the LDT on **A**) time (sec ± S.E.M., compared to the total duration of the test of 300 sec) spent in the light compartment and in the dark compartment, latency to enter the dark compartment, rearing (time spent standing on the hind legs), and risk assessment (time in which incomplete head and/or body extends that dips from the dark to the light compartment); **B**) number (average of the number of times ± S.E.M.) of transitions between compartments and risk assessment in CTRL, HFD, and HFD + DZ 2w hamster groups. Statistical differences were evaluated by using a two-way ANOVA followed by post hoc Newman-Keul’s multiple range test when *p*-value < 0.05. *p < 0.05, **p < 0.01, ***p < 0.001. *Significant difference compared with CTRL group; a, b, c Significant difference of HFD + DZ 2w compared with HFD group

HFD hamsters treated with DZ displayed improved body weight with respect to CTRLs, as indicated in our previous work [5]. At the same time, they exhibited anxiety-like behaviors in LDT exploration paradigm, which were attenuated in the presence of DZ. In particular, the different behavioral evaluations (*F*_(2,15)_ = 3.69; *p* < 0.05) resulted to be reduced in HFD hamsters as indicated by moderate diminished levels of permanence in light compartment (− 45%) and of rearing (− 33%) with respect to CTRLs (Fig. 1A). Conversely, HFD hamsters treated with DZ for 2 w spent more time (+ 51%) in the light compartment with respect to CTRLs and this turned out to be extremely higher when compared to HFD hamsters (+ 147%; Fig. 1A). In addition, HFD + DZ 2w hamsters explicated more transitions between the two compartments (+ 200%) while contemporarily reducing both the time (− 66%; Fig. 1B) and the number of times (− 43%) of risk assessment.

**Fig. 2:**
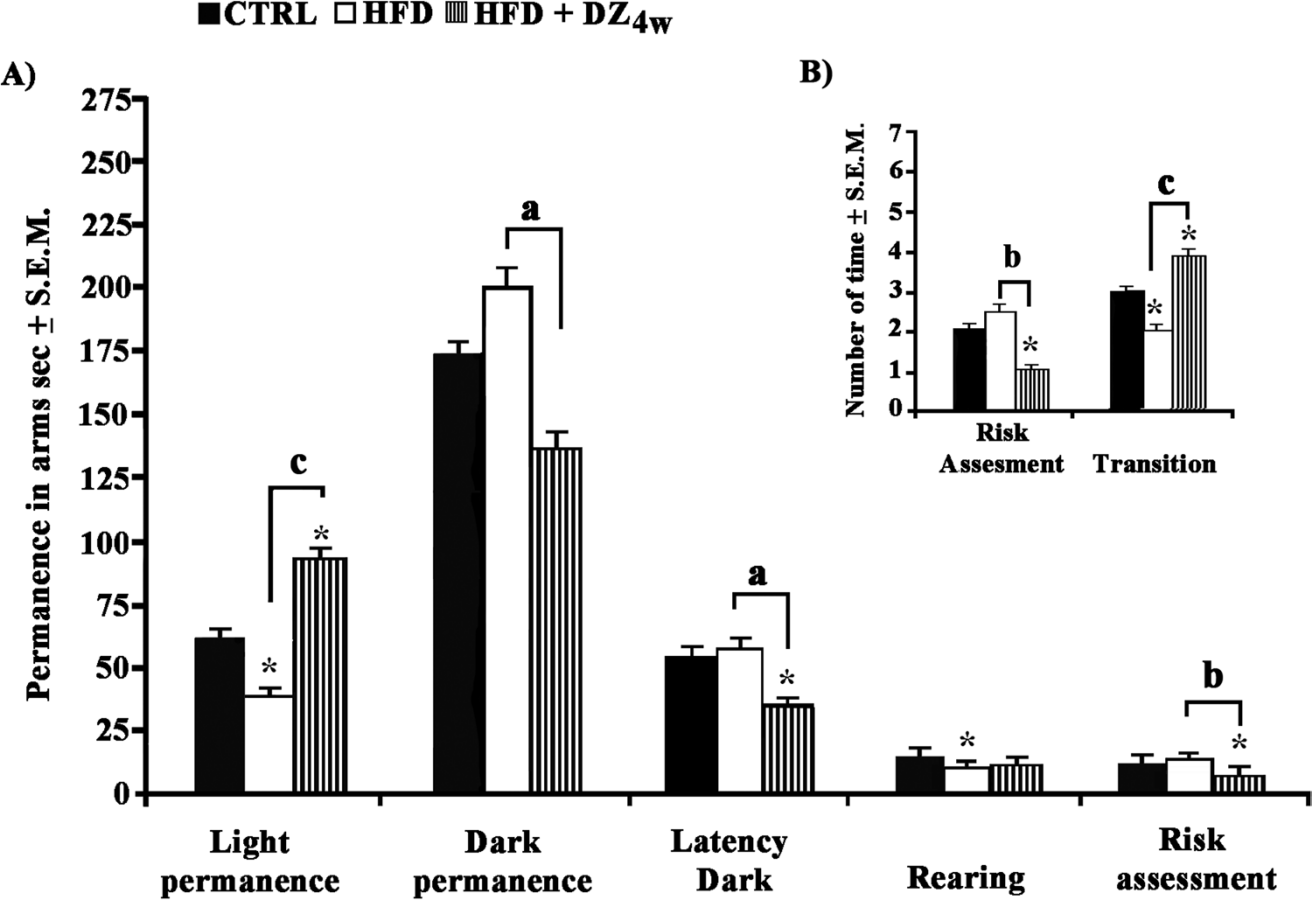
Evaluation of the anxiolytic activity of DZ using the LDT on** A**) time (sec ± S.E.M., compared to the total duration of the test of 300 sec) spent in the light compartment and in the dark compartment, latency to enter the dark compartment, rearing, and risk assessment; **B**) number (average of the number of times ± S.E.M.) of transitions between compartments and risk assessment-number in CTRL, HFD, and HFD + DZ 4w hamster groups. Statistical calculations were handled as reported in Fig. 1

It was worthy to note that HFD hamsters after 4 w of DZ treatment (Fig. 2A and B) continued to exhibit similar but somewhat greater behavioral trends (*F*_(2,15)_ = 3.75; *p* < 0.05). Even in this case, HFD hamsters spent less time (− 54%) in light compartment along with showing a diminished rearing activity (− 48%), when compared to CTRLs while despite HFD + DZ hamsters having spent extremely more time in the light compartment (+ 215%), displayed a reduction of both permanence (− 60%) and number of risk assessment with respect to HFD hamsters. Moreover, HFD + DZ4w animals exhibited extremely more transitions between the two compartments with respect to HFD hamsters (+ 100%) whereas it was not of the same entity when compared to CTRLs (+ 33%). Additionally, a moderate decrease (− 49%) in the first latency to enter the dark compartment was also detected.

### Evaluation of Locomotor Activity in NCT

**Fig. 3:**
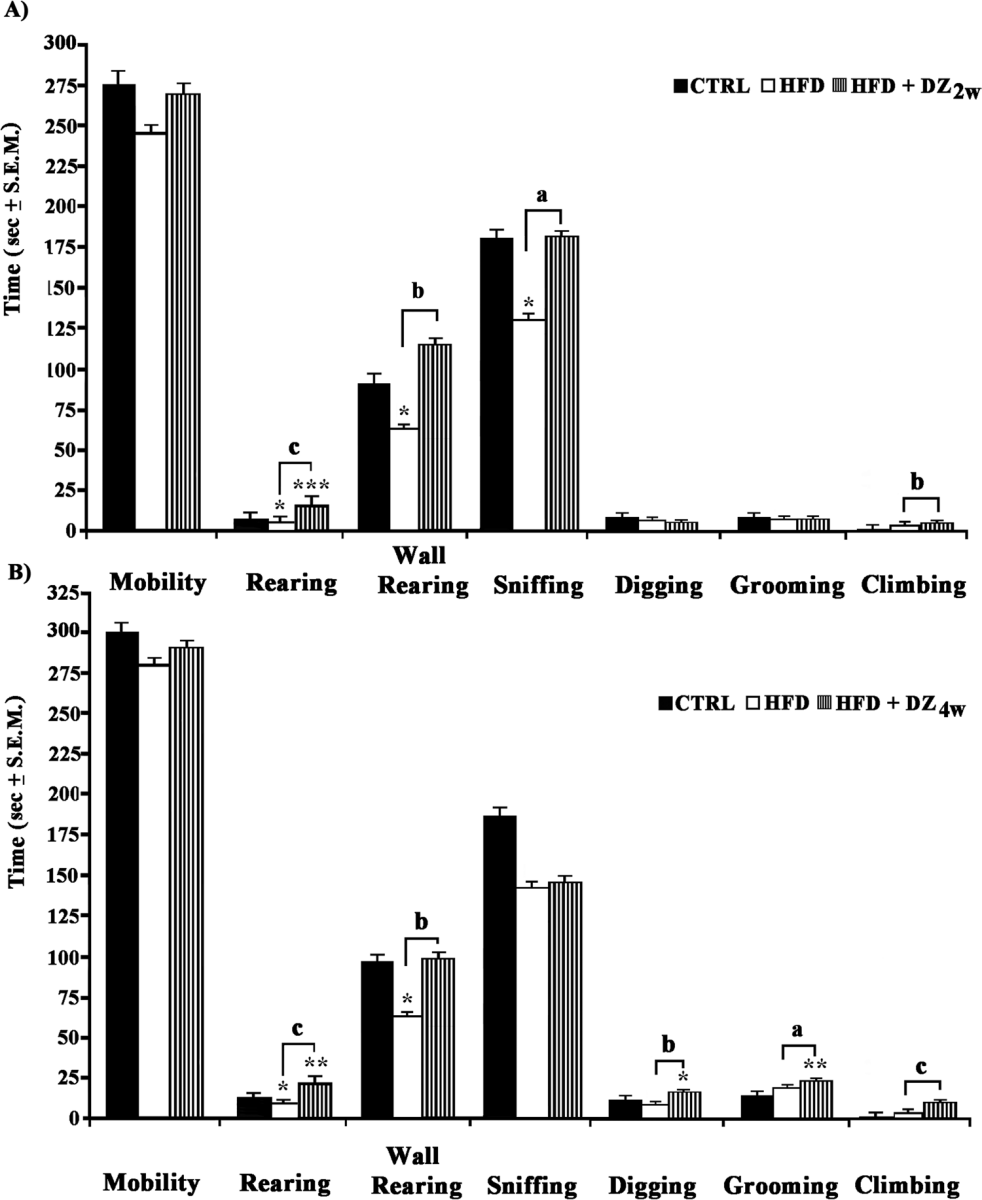
Evaluation of the spontaneous locomotor activity in **A**) CTRL, HFD, and HFD + DZ 2w and **B**) CTRL, HFD, and HFD + DZ 4w hamster groups using the NCT on time (sec ± S.E.M. compared to the total duration of the test of 300 sec) spent in: mobility (movement in the cage), rearing (standing on hind limbs), wall-rearing (standing on hind limbs and touching the walls of the cage with forelimbs), sniffing (sniffing sawdust), digging (digging sawdust with forelimbs, often kicking it away with hind limbs), grooming (licking own fur, sometimes using forepaws, passing them over the nose with a series of brief, horizontal movements), and climbing (escape behavior). Statistical calculations were handled as reported in Fig. 1

For analyzing further spontaneous exploration activity in a non-aversive environment (cage), NCT was also evaluated. In this case a low reactivity (*F*_(2,15)_ = 3.87; *p* < 0.05) appeared to be induced by HFD as suggested by HFD group spending less time (~ − 33%) performing rearing, wall rearing (Fig. 3A and B) and sniffing (Fig. 3B) behaviors with respect to CTRLs. Conversely, HFD + DZ2w hamsters spent more time in rearing (+ 200%) when compared to CTRLs, while on the other hand performed more wall rearing plus climbing (~ + 60%) together with a moderate sniffing activity (+ 42%), this time with respect to HFD. Such an effect widely improved (*F*_(2,15)_ = 6. 90; p < 0.01) after a 4-week DZ diet (Fig. 3B) as indicated by an extremely evident rearing (+ 270%) and climbing (+ 225%) activities when compared to the HFD group hamsters. These animals also displayed high dipping (+ 62%) and wall rearing (− 41%) along with a moderate grooming activity (− 33%), which proved to be more frequent than in HFD hamsters.

### Effects of DZ on Cerebellar TrkB Expression

**Fig. 4:**
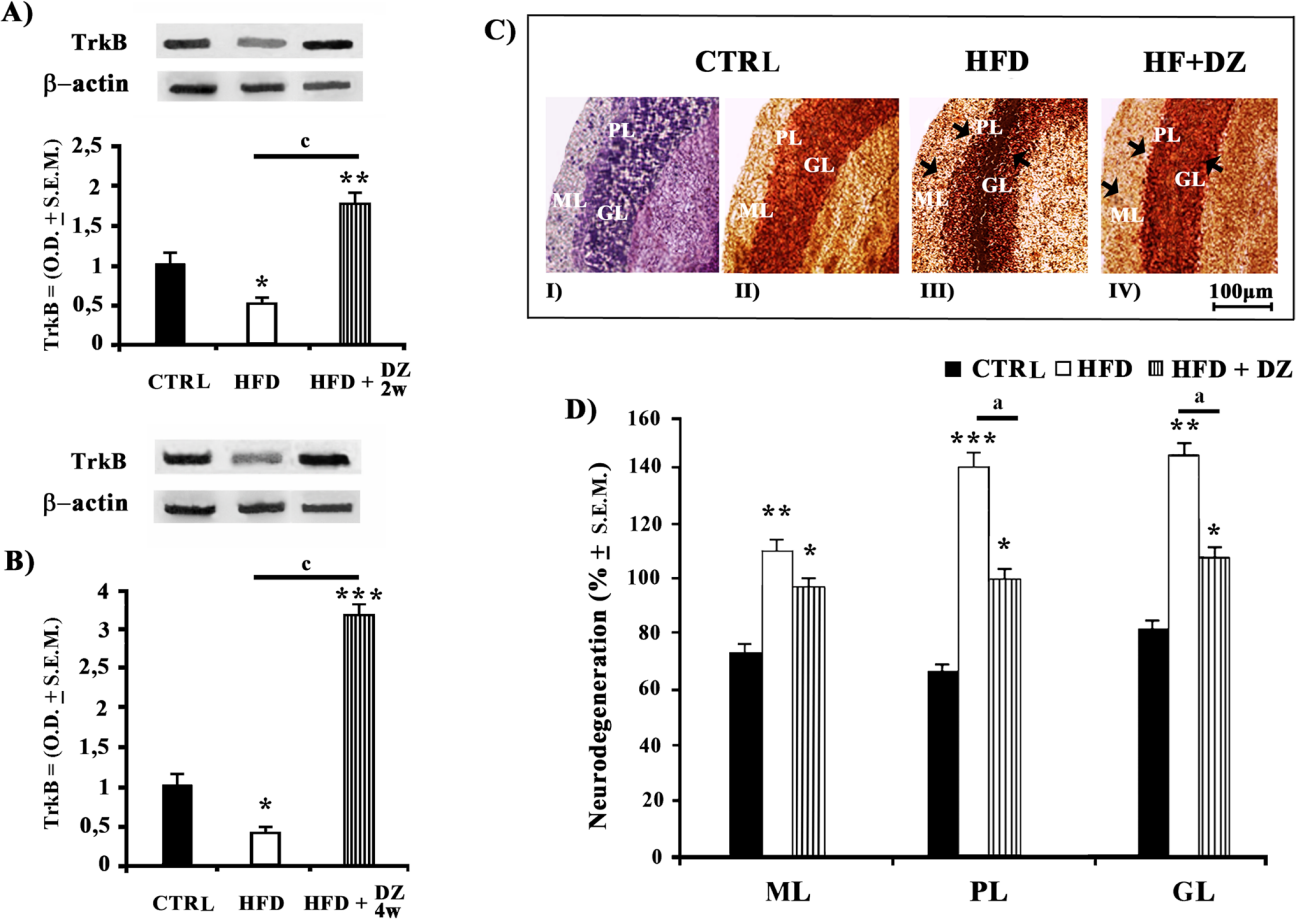
Western blot analysis of TrkB expression (O.D. ± S.E.M.) in cerebellar cortex of **A**) CTRL, HFD, and HFD + DZ 2w and **B**) CTRL, HFD, and HFD + DZ 4w hamsters. **C**) Representative image of coronal cerebellar cortex sections stained with (I) Nissl and (II; III; IV) ACS. Photograms of ACS showing dark damaged neuronal perikarya that indicate the different levels of neurodegeneration (arrows; Scale bar = 100 µm). **D**) Estimation of neurodegeneration (% ± S.E.M.) of degenerated neurons in ML, PL, and GL of HFD, and HFD + DZ 4w groups with respect to CTRL. Statistical calculations were handled as reported in Fig. 1. Abbreviations: ML, molecular layer; PL, Purkinje layer; GL, granular layer

The expression of TrkB protein in the cerebellum of HFD hamsters resulted to be moderately downregulated (− 50%; *p* < 0.05) with respect to CTRLs (Fig. 4A and B), while a notable up-regulation was instead reported for HFD + DZ2w (+ 75%; *p* < 0.01). The increment of TrkB levels turned out to be extremely higher (+ 220%) in HFD + DZ2w when compared to HFD hamsters. Furthermore, and in a very evident fashion, hamsters treated with DZ continued to improve the largely reduced expression levels of TrkB in HFD especially after 4 weeks of diet (Fig. 4B) as indicated by its extremely elevated up-regulation with respect to CTRL (+ 200%) and this up-regulation resulted to be even greater (+ 700%) when the expression levels of HFD + DZ were compared, this time to those of HFD hamsters.

### Effects of DZ on Plasma Lipid Profiles

From the plasma lipidic differences in HFD group (Table 1), it appeared that TC levels were significantly increased (+ 130%), with a very evident (+ 190%) and moderate (+ 33%) increase of LDL and HDL levels, respectively, than CTRLs. In contrast, HFD + DZ 4w hamsters appeared to supply a somewhat consistent reduction (− 74%) of the harmful cholesterol component (LDL) together with a moderate reduction (− 35%) of TC.

**Table 1:** Plasma total cholesterol (TC), LDL cholesterol and HDL cholesterol levels (mg/dl ± S.E.M.) of HFD hamster ± DZ4w with respect to both CTRL (*) and to group exposed to HFD alone (letters). Statistical calculations were handled as reported in the Fig. legend 1

mg/dl ± SEM	CTRL	HFD	HFD + DZ
HDL cholesterol	63 ± 7	84 ± 11*	96 ± 9
LDL cholesterol	89 ± 9	258 ± 26***	67 ± 7^**b**^
TC (cholesterol)	117 ± 12	270 ± 28***	175 ± 20^**a**^

### Cerebellar Histological Changes Following HFD and DZ Treatments

From the histological analyses of the cerebellar cortical layers in CTRLs stained with Nissl (Fig. 4C, I) as well as for ACS labeled sections (Fig. 4C, II), it was possible to observe specific neuronal damages in the different cerebellar regions. In this case, the cerebellar cortex of HFD hamsters exhibited altered neuronal histological conditions as indicated by the evident argyrophilic reaction (Fig. 4C, III) that supplied dense dark granules in the molecular (ML), Purkinje cell (PL), and granular (GL) layers. In particular, evident neuronal damages were detected (Fig. 4D) in the Purkinje cell layer (PL; + 103%) of HFD hamsters with respect to CTRLs. As for the other regions, only a significantly high density of granules was observed in the molecular (ML; + 62%) and granular layers (GL; + 78%). Conversely, the argyrophilic reaction in HFD hamsters fed with DZ for 4 weeks was moderately reduced (~ 30%) in the same above cortical layers with respect to CTRLs. An even stronger reduction of the dark granules seemed to characterize PL (− 30%) and GL (− 42%) layers of HFD 4w-treated hamsters with respect to HFD group, if we consider the heavily dense levels in obese hamsters with respect to CTRLs.

## Discussion

The aim of the present study was to examine the role of HFD, separately, and in combination with DZ towards behavioral performances along with indications, obtained for the first time on the expression levels of the high-affinity catalytic receptor for several neurotrophins in the hamster cerebellar cortex. The results tend to corroborate an evident effect of HFD not only towards exploratory and anxiety-related behaviors but also for expression levels of TrkB in the cerebellar cortex. It was noteworthy that the altered expression levels of this neurotrophic factor appeared to be tightly related with neuronal death in the cortical layers thus supporting the hypothesis that the cerebellum may turn out to be a pivotal station controlling emotional reactions [29] as well as being involved with the explication of neurodegenerative mechanisms occurring in states of obesity [30].

From a behavioral point of view, HFD hamsters rapidly displayed a reduction of spontaneous locomotor behavior as indicated by a decrease in behavioral reactivity, e.g., decreased time of rearing and wall rearing, when hamsters were placed in a novel clean cage during NCT, which was similar to that reported for HFD 57BL/6 J mice [31]. It was not unusual to observe altered explorative performances concomitantly with other anxiety-related motor activities in HFD treated animals as suggested by a decrease of time spent in the light chamber of the LDT box considered the aversive site [32]. However, when HFD hamsters were treated with DZ, an inverse situation of HFD-induced behavioral performances occurred in both NCT plus LDT apparatus, which resulted to be in line with other polyphenols reducing anxiety states and restoring spontaneous locomotor activity and exploration-like behaviors in obese rats [33]. In this context, it appears that the potential role of dietary phytoestrogens towards the restoration of explorative behaviors in obese animals [29] may be accomplished via its anxiolytic-like effects [34] due to reduced depressive-like behaviors plus enhanced synaptic plasticity processes [35] occurring above all in motor-controlling brain regions.

The recovery of behavioral activities by phytoestrogens may also be due to their ability of re-establishing lipidic levels as suggested by the accumulating data on dietary polyphenols playing a vital role on lipoprotein metabolism [36, 37]. Such feature seems to derive from the antioxidant as well as anxiolytic properties of phytoestrogens [34], despite the mechanisms by which these compounds regulate behavioral changes in the presence of reduced lipidic levels in obesity conditions have not yet been fully elucidated. In our work, the various behavioral alterations in HFD hamsters like those provided by other studies [5, 38, 39] have shown to be related to elevated hyperlipidemia levels [40, 41]. This feature appears to go in the same direction of elevated total cholesterol and LDL levels exhibited also in our obese animal model, conditions which are tightly linked with neurodegenerative syndromes like Alzheimer’s disease or other atypical cognitive deficits [42, 43].

Interestingly, the variations of our behavioral data seemed to be tightly correlated to decreased expression of TrkB in the cerebellum, brain site that is known to be, via reciprocal connections with the prefrontal cortex, not only a key motor controlling site but recently has been also shown to influence appetite signals in obesity conditions [44]. The importance of the neurotrophic factor in the cerebellum is further supported by this major neurotrophic factor, acting as a mitogenic and chemotactic factor, after birth, stimulating the cerebellar cell precursors of GL to proliferate, migrate, and maturate along with the dendritic development of PL plus being highly expressed in such a brain region of adults [45]. Indeed, visceral adiposity in obese animals has shown to modify cerebellar morphological-functional changes that consequently interfere with motor, cognitive, and emotional processes [46]. It has also been shown that obesity and psychosocial stress tend to account for a heavy toll on the bioavailability of BDNF that is required for its adequate interaction with TrkB receptor and thus assuring an appropriate regulatory role on neuroplasticity events necessary for neuronal cells to cope with stressful conditions [47]. The highlighting, for the first time, of anxiety-like and altered spontaneous locomotor behaviors in LDT and NCT being related to low cerebellar expression levels of TrkB in HFD hamsters may bring us closer to the understanding of altered motor activities in obesity states. Indeed, the ameliorated expression of BDNF has been shown to occur in several neurodegenerative diseases, including cerebellar pathologies such as spinocerebellar ataxia type 6 [48], schizophrenia, and bipolar disorder state [49, 50]. Thus, TrkB changes in cerebellum may result to be either a cause or a consequential effect of obesity thereby enhancing a dysregulation capacity in exploratory and anxiety behaviors. Under such a condition, DZ might be inducing anxiolytic-like effects because this phytoestrogen by interacting with β estrogen receptor site tends to exert a restoration effect not only for anxiolytic states deriving from altered levels of this estrogenic site but more importantly for environmental exploration performances [51]. In this context, the increased cerebellar levels of TrkB in HFD-fed hamsters after dietary addition of DZ may constitute another element underlying the importance of this brain station operating under anxiety-like conditions. Furthermore, the reduction of HFD-induced neuronal death in cerebellar cortex, which is evident 16 weeks after diet, may represent a crucial step concerning the involvement of DZ on the restoration of the cerebellar cortical areas through neurotrophic-dependent neuronal plasticity events in HFD-induced brain impairments [52, 53]. At the same time, DZ-dependent TrkB protective effects in the cerebellum propose this brain region as a key site for the recovery of motor-related behavioral functions especially in obesity conditions.

## Conclusion

In summary, our data support the important role played by all three cortical layers of the cerebellum in obese hamsters as suggested by the altered locomotor plus anxiety-like responses as well as being involved with neurodegenerative events during such a syndrome. Following the administration of DZ, hamsters rapidly displayed an evident preference for the light compartment, plus transiting more frequently between the two compartments as well as reduced spontaneous exploration in a non-aversive environment. Treatment with this phytoestrogen also pointed to an extremely elevated increase of TrkB expression in cerebellum cortex thus inverting the effects caused by obesity conditions and namely inflammatory-induced depression-like behaviors [54]. Overall, these first neuroprotective effects of DZ in the cerebellar layers strongly corroborate its major role in altered locomotor and emotional behavioral performances and this may open new interests on how the manipulation of BDNF and its main intracellular signaling mechanisms may contribute to the introduction of new and innovative drug strategies for treating such neurodegenerative syndromes.
